# Interaction between anemia and hyperuricemia in the risk of all-cause mortality in patients with chronic kidney disease

**DOI:** 10.3389/fendo.2024.1286206

**Published:** 2024-03-22

**Authors:** Zhaoxuan Lu, Fangping Lu, Ruixue Zhang, Shuting Guo

**Affiliations:** ^1^ Blood Purification Center, Beijing Puren Hospital, Beijing, China; ^2^ Department of Nephrology, The First Hospital of Tsinghua University, Beijing, China; ^3^ Department of Rheumatology, The First Hospital of Tsinghua University, Beijing, China

**Keywords:** anemia, hyperuricemia, chronic kidney disease, all-cause mortality, NHANES

## Abstract

**Aim:**

Both hyperuricemia and anemia are not only the manifestation of chronic kidney disease (CKD) but also related to its occurrence and development. A recent study has found that there was a synergetic effect between hyperuricemia and anemia on new-onset CKD. Herein we aimed to explore the roles of hyperuricemia and anemia in the all-cause mortality in patients with CKD.

**Methods:**

Data of adult patients with CKD were extracted from the National Health and Nutrition Examination Surveys (NHANES) database in 2009–2018 in this retrospective cohort study. Weighted univariate and multivariate COX regression analyses were used to investigate the associations of hyperuricemia and anemia with all-cause mortality, and the evaluation indexes were hazard ratios (HRs) and 95% confidence intervals (CIs). The interaction effect between hyperuricemia and anemia on the risk of all-cause mortality was assessed via relative excess risk due to interaction (RERI) and attributable proportion of interaction (AP). Subgroup analyses of age, gender, CVD, hypertension, DM, and cancer were also performed to assess this interaction effect.

**Results:**

Among 3,678 eligible patients, 819 died from all causes. After adjusting for covariables, we found that CKD patients with anemia (HR = 1.72, 95%CI: 1.42–2.09) or hyperuricemia (HR = 1.21, 95%CI: 1.01–11.45) had a higher risk of all-cause mortality. There was a potential synergetic effect between anemia and hyperuricemia on all-cause mortality, with RERI of 0.630 and AP of 0.291. Moreover, this synergetic effect was also observed in ≥65 years old (AP = 0.330), male (AP = 0.355), hypertension (AP = 0.736), non-hypertension (AP = 0.281), DM (AP = 0.371), and cancer (AP = 0.391) subgroups.

**Conclusion:**

A potential synergetic effect between anemia and hyperuricemia on all-cause mortality was found in patients with CKD. However, further studies are needed to clarify the causal relationship between them.

## Introduction

Chronic kidney disease (CKD) is one of the progressive diseases affecting approximately 13% of the world’s population (1). CKD has a significant impact on morbidity, mortality, and quality of life worldwide (2, 3), and about 15% of adults in the United States have CKD according to the statistics of the Centers for Disease Control and Prevention (CDC) (4). Compared with persons who have normal kidney functions, those who with CKD had a higher risk of all-cause mortality, which resulted in a severe disease burden (5). Hence, the accurate identification of influencing factors for CKD prognosis is of great significance for implementing reasonable intervention as well as reducing the disease burden.

Uric acid is the end product of nucleic acid metabolism and is also generated during the breakdown of high-energy nucleotides (6, 7). In humans, excretion of uric acid is primarily via the kidney (two-thirds of total elimination) and gut (one-third) (8). As kidney function declines, uric acid is retained, leading to a relative increased excretion of uric acid both through glomerular filtration and the extra-renal pathway (e.g., intestinal excretion) (9, 10). In fact, hyperuricemia has been reported to be not only the manifestation of CKD but also associated with the occurrence and development of CKD (11). Uric acid may play a role in activating lipid oxidation mediated by peroxynitrite, stimulating pro-inflammatory biomarkers, and reducing nitric oxide endothelium levels, thus contributing to the progression of CKD (12, 13). Epidemiological evidences have suggested that elevated serum uric acid concentration was linked to a higher risk of all-cause mortality in patients with CKD (14, 15). In addition to hyperuricemia, anemia is also a common complication of CKD, and its prevalence increases with the exacerbation of CKD (16). Similar to the effects of hyperuricemia, anemia can also induce inflammation and oxidative stress in the extracellular matrix and endothelial cells, participating in the progression of CKD (17, 18). A recent large-sample cohort study in Taiwan found that hyperuricemia and anemia were related to new-onset CKD, respectively, and also had a synergetic effect on new-onset CKD (19). However, the interaction effect between hyperuricemia and anemia on CKD prognosis has not been discussed.

This study aims to investigate herein the associations of hyperuricemia and anemia with all-cause mortality in patients with CKD and discuss whether there is a synergetic effect between hyperuricemia and anemia. We hope that this study may provide some information for risk stratification management and the formulation of intervention measures among CKD patients.

## Methods

### Study design and participants

This retrospective cohort study was based on the National Health and Nutrition Examination Surveys (NHANES) database (2009–2018). The NHANES, with the aim of assessing the nutritional and health status of noninstitutionalized population in the United States, is conducted by the Centers for Disease Control and Prevention (CDC) as well as the National Center for Health Statistics (NCHS). The survey uses a complex and multistage stratified probability sampling from selected counties, blocks, households, and persons within households. Interviews in participants’ homes were conducted by NCHS well-trained professionals, and the extensive physical examinations were conducted at mobile exam centers (MECs). More details are accessible on the NAHENS website: https://www.cdc.gov/nchs/nhanes/index.htm.

Initially, 4,642 adult patients with CKD in the database were included. Those who were without information on serum hemoglobin (HB) or uric acid or had an extreme energy intake (<500 or >8,000 kcal in male patients and <500 or >5,000 kcal in female patients) were excluded. After excluding those who were lost to follow-up, 3,678 patients were finally considered eligible. Since the NHANES database is publicly available and all data have been de-identified, the ethical approval has been waived from the Institutional Review Board (IRB) of Beijing Puren Hospital.

### Assessment of chronic kidney disease, hyperuricemia, and anemia

According to the “Kidney Disease: Improving Global Outcomes (KDIGO) 2021 Guidelines”, urinary albumin to creatinine ratio (UACR) >30 mg/g and/or estimated glomerular filtration rate (eGFR) <60 mL/min/1.73 m^2^ were identified as CKD (20). The eGFR was calculated using the following formula: eGFR = 175 × standardized serum creatinine (Scr) - 1.154 × age - 0.203 × 1.212 (if black) × 0.742 (if female), where GFR is expressed as mL/min/1.73 m^2^ of body surface area and Scr is expressed in mg/dL.

Hyperuricemia was defined as a serum uric acid level >7 mg/dL in male patients and >6.0 mg/dL in female patients (21). Moreover, the definition of anemia was based on serum HB level (<13 mg/dL in male patients and <12 mg/dL in female patients), which values are recommended by the World Health Organization (WHO) (22).

### Outcome and follow-up

The study outcome was all-cause mortality. The determination of mortality was on the basis of a probability matching algorithm by the National Death Index (NDI) and using the public-use NHANES-linked mortality file correlated with the NCHS. The follow-up ended until the patients died or on December 31, 2019. The details are shown elsewhere: (https://ftp.cdc.gov/pub/health_statistics/NCHS/datalinkage/linked_mortality/).

### Selection of variables

We extracted variables that could be potential covariates from the database, including age, gender, race, education level, marital status, poverty income ratio (PIR), smoking, drinking, physical activity, hypertension, dyslipidemia, cardiovascular disease (CVD), diabetes mellitus (DM), cancer, body mass index (BMI), eGFR, UACR, white blood cell (WBC), lymphocyte, neutrophil, platelet, Mediterranean (MED) score, protein supply ratio, carbohydrate supply ratio, total fat supply ratio, HB, uric acid, serum vitamin D, serum sodium (Na), transferrin saturation (TSAT), and ferritin.

The NHANES collected the blood samples of participants through physical examinations by trained professionals at MECs. They were then divided into aliquots and shipped to multiple laboratories for further analysis. The laboratory procedures and quality control methods for the measurement of serum indexes were described in detail on the NCHS website: https://www.cdc.gov/nchs/nhanes. In this study, BMI was divided into three categories according to the WHO criteria: underweight/normal (<18.5 kg/m^2^/18.5–24.9 kg/m^2^), overweight (25–29.9 kg/m^2^), and obesity (≥30 kg/m^2^).

Demographic and behavioral information was collected via the NHANES household interviews using different questionnaires. Individuals who had self-reported to have smoked more than 100 cigarettes in their lives were recognized as smokers. Alcohol consumption was divided into three levels (excessive drinking, light drinking, and never drinking). Physical activity was converted to metabolic equivalent (MET) using the following formula: energy expenditure (MET·min) = recommended MET × exercise time of corresponding activity (min).

Hypertension, dyslipidemia, CVD, DM, and cancers were diagnosed based on laboratory tests, self-reported medical history, or medication history. The consumptions of total energy, protein, carbohydrate, and total fat were calculated by “dietary intake plus dietary supplements”. The calculations for protein supply ratio, carbohydrate supply ratio, and total fat supply ratio were on the basis of total energy intake as well as their respective intake: protein supply ratio = protein intake × 4/total energy intake × 100%; carbohydrate supply ratio = carbohydrate intake × 4/total energy intake ×100%; and total fat supply ratio = fat intake × 9/total energy intake × 100%. Hyponatremia was defined as serum Na concentration <135 mmol/L, while the definition of hypernatremia was serum Na concentration ≥145 mmol/L (23). The cutoff values of TSAT (20%) and ferritin (100 mg/mL) were according to a previous study (24).

### Statistical analysis

Normally distributed data were described by mean ± standard error (mean ± SE), and *t*-test was used for a comparison between the two groups. Categorical data were expressed as frequency with constituent ratio [N (%)], and chi-square test (*χ* ^2^) was employed for the comparison. The NHANES special weights “the two-year sample weights (WTMEC2YR)” were used in this study. Detailed instructions for combining datasets from the NHANES cycles are provided in the NHANES Analytic Guidelines: https://wwwn.cdc.gov/Nchs/Nhanes/2011-2012/DEMO_G.htm.

Weighted univariate COX regression (**Supplementary Table S2**, model 1) and stepwise regression (**Supplementary Table S2**, model 2) analyses were used to screen the covariates. Then, weighted univariate and multivariate COX regression analyses were utilized to investigate the associations of hyperuricemia and anemia with all-cause mortality. The univariate model did not adjust for covariates, whereas the multivariate model adjusted for covariates which associated with all-cause mortality selected via the above-mentioned methods (with *P* < 0.05), including age, gender, race, marital status, PIR, smoking, physical activity, hypertension, CVD, DM, cancer, and carbohydrate supply ratio. Furthermore, we explored these relationships in subgroups of age, gender, CVD, hypertension, DM, and cancer. The covariates adjusted in multivariate models were those mentioned above (except the variables used to stratify in each subgroup). The evaluation indexes were hazard ratios (HRs) and 95% confidence intervals (CIs). Two-sided *P <*0.05 was considered statistically significant.

We further assessed the interaction effect between hyperuricemia and anemia on the risk of all-cause mortality through measuring whether the estimated joint effect of the two factors was greater than the sum of the independent effect of serum hyperuricemia and anemia. Relative excess risk due to interaction (RERI) and attributable proportion of interaction (AP) were utilized to evaluate the interaction effect. When the confidence interval of RERI and AP contained 0, there was no interaction effect. In addition, we drew Kaplan–Meier (KM) curves to reflect the survival probability of patients with/without hyperuricemia or anemia as well as their interaction effect on all-cause mortality.

Statistical analysis was performed using SAS 9.4 (SAS Institute, Cary, NC, USA). Variables with missing data (including lymphocyte, neutrophil, BMI, and PIR) were interpolated using random forest method. The sensitivity analysis for the characteristics of participants before and after the missing data’s interpolation is shown in **Supplementary Table S1**.

## Results

### Characteristics of participants

**Figure 1:**
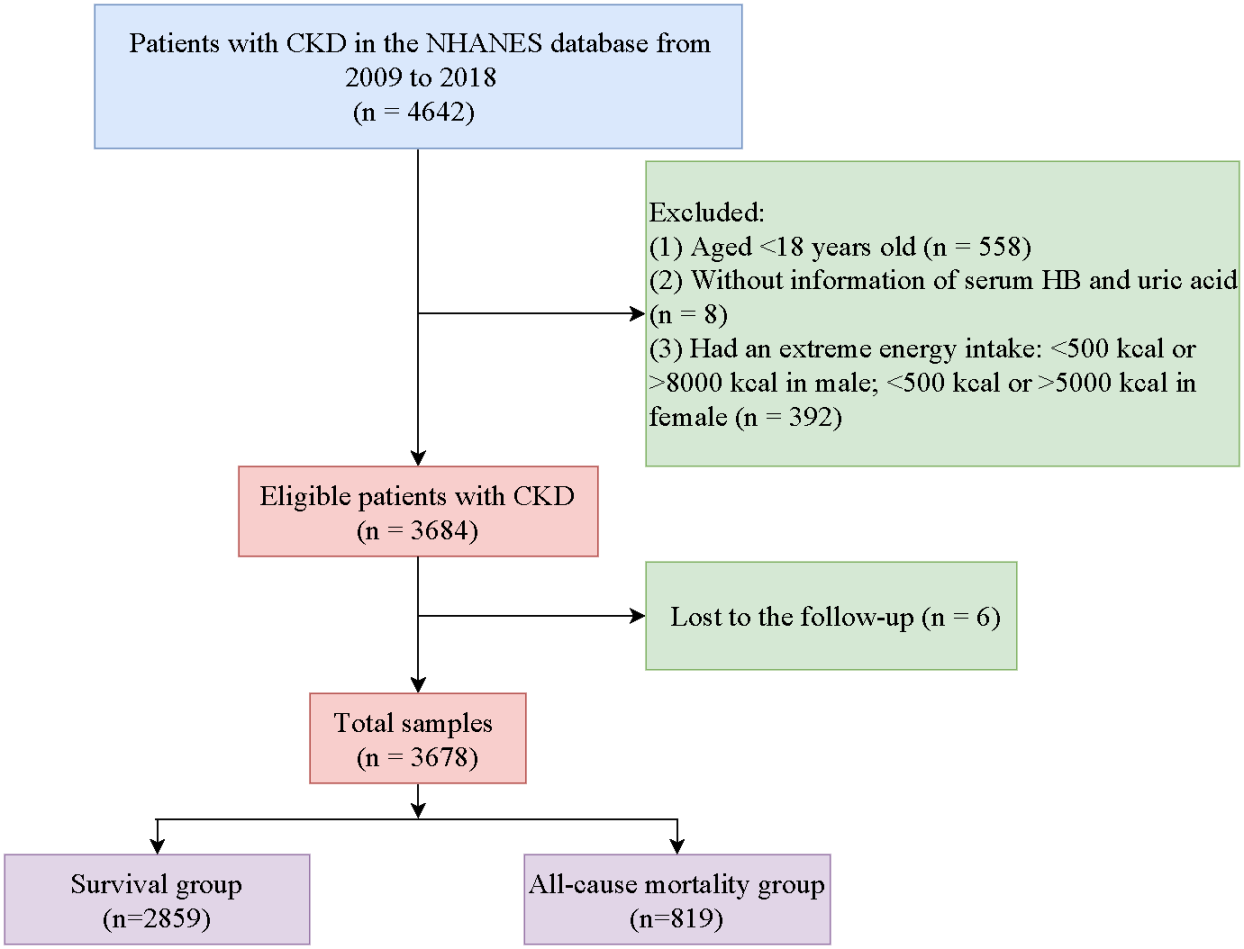
Flowchart of the screening of participants.

**Figure 1** shows a flowchart of the screening of study participants. Initially, we included 4,642 patients with CKD from the NHANES. Then, we excluded those aged <18 years old (*n* = 558), without information on serum HB or uric acid (*n* = 8), or having an extreme energy intake (*n* = 392). Moreover, patients lost to follow-up were excluded (*n* = 6). Finally, 3,678 patients were deemed eligible.

The characteristics of individuals between the survival group (*n* = 2,859) and the all-cause mortality group (*n* = 819) were compared. The average follow-up time was 63.82 months, and among the total number of patients, only 21 (0.5%) died due to renal disease. The mean age of the participants was 58.98 years old, and 1,855 (53.93%) of them were female. Among CKD patients, 802 (16.5%) had anemia, whereas 1,218 (32.71%) had hyperuricemia. More details on the participants’ characteristics are shown in **Table 1**.

**Table 1:** Characteristics of patients with CKD.

Variables	Total (*n* = 3,678)	Survival (*n* = 2,859)	All-cause mortality (*n* = 819)	Statistics	*P*
Age, years, mean (SE)	58.98 (0.45)	55.98 (0.50)	71.55 (0.46)	*t* = -23.62	**<0.001**
Age, years, *n* (%)				*χ* ^2^ = 174.047	**<0.001**
<65	1,801 (52.97)	1,648 (60.07)	153 (23.17)		
≥65	1,877 (47.04)	1,211 (39.93)	666 (76.83)		
Gender, *n* (%)				*χ* ^2^ = 6.171	**0.013**
Female	1,855 (53.93)	1,512 (55.10)	343 (49.04)		
Male	1,823 (46.07)	1,347 (44.90)	476 (50.96)		
Race, *n* (%)				*χ* ^2^ = 53.948	**<0.001**
Black	829 (12.45)	678 (13.12)	151 (9.63)		
White	1,609 (66.45)	1,103 (63.54)	506 (78.66)		
Others	1,240 (21.10)	1,078 (23.34)	162 (11.71)		
Education level, *n* (%)				*χ* ^2^ = 39.123	**<0.001**
High school graduate or below	1,935 (45.35)	1,440 (43.53)	495 (52.95)		
Some college or above	1,652 (52.70)	1,333 (54.13)	319 (46.71)		
Unknown	91 (1.95)	86 (2.34)	5 (0.33)		
Marital status, *n* (%)				*χ* ^2^ = 18.381	**<0.001**
Married	1,780 (51.75)	1,401 (52.35)	379 (49.23)		
Not married	1,809 (46.33)	1,373 (45.35)	436 (50.48)		
Unknown	89 (1.91)	85 (2.30)	4 (0.30)		
PIR, mean (SE)	2.62 (0.05)	2.69 (0.06)	2.33 (0.07)	*t* = 4.53	**<0.001**
Smoking, *n* (%)				*χ* ^2^ = 28.695	**<0.001**
No	1,863 (50.43)	1,530 (52.48)	333 (41.82)		
Yes	1,769 (48.58)	1,288 (46.39)	481 (57.73)		
Unknown	46 (1.00)	41 (1.13)	5 (0.44)		
Drinking, *n* (%)				*χ* ^2^ = 3.506	0.173
Excessive	325 (11.22)	262 (11.87)	63 (8.45)		
Light	292 (9.23)	226 (9.34)	66 (8.76)		
Never	3,061 (79.56)	2,371 (78.79)	690 (82.79)		
Physical activity, MET·min, *n* (%)				*χ* ^2^ = 130.545	**<0.001**
<450	1,035 (29.04)	830 (30.00)	205 (25.03)		
≥450	1,221 (36.50)	1,075 (40.76)	146 (18.62)		
Unknown	1,422 (34.46)	954 (29.24)	468 (56.34)		
Hypertension, *n* (%)				*χ* ^2^ = 76.916	**<0.001**
No	669 (22.23)	610 (25.89)	59 (6.86)		
Yes	3,009 (77.78)	2,249 (74.11)	760 (93.14)		
Dyslipidemia, *n* (%)				*χ* ^2^ = 27.212	**<0.001**
No	686 (19.38)	571 (21.08)	115 (12.26)		
Yes	2,992 (80.62)	2,288 (78.92)	704 (87.74)		
CVD, *n* (%)				*χ* ^2^ = 154.278	**<0.001**
No	2,010 (59.05)	1,746 (64.65)	264 (35.60)		
Yes	1,668 (40.95)	1,113 (35.36)	555 (64.40)		
DM, *n* (%)				*χ* ^2^ = 38.529	**<0.001**
No	2,198 (65.13)	1,775 (67.73)	423 (54.27)		
Yes	1,480 (34.87)	1,084 (32.28)	396 (45.73)		
Cancer, *n* (%)				*χ* ^2^ = 78.399	**<0.001**
No	2,972 (80.36)	2,376 (82.58)	596 (71.05)		
Yes	613 (17.67)	394 (15.05)	219 (28.65)		
Unknown	93 (1.97)	89 (2.37)	4 (0.30)		
BMI, kg/m^2^, mean (SE)	30.44 (0.19)	30.50 (0.21)	30.19 (0.34)	*t* = 0.81	0.422
BMI, *n* (%)				*χ* ^2^ = 2.593	0.274
Obesity	1,674 (46.79)	1,346 (47.45)	328 (44.01)		
Overweight	1,159 (29.84)	884 (29.76)	275 (30.16)		
Underweight/normal	845 (23.38)	629 (22.79)	216 (25.83)		
eGFR, mL/min/1.73 m^2^, mean (SE)	82.80 (0.65)	87.15 (0.76)	64.57 (1.20)	*t* = 15.19	**<0.001**
UACR, mg/g, mean (SE)	197.46 (12.64)	179.93 (13.12)	270.96 (33.92)	*t* = -2.54	**0.013**
WBC, 1,000 cells/uL, mean (SE)	7.59 (0.06)	7.54 (0.06)	7.79 (0.15)	*t* = -1.55	0.125
Lymphocyte, 1,000 cells/uL, mean (SE)	2.07 (0.03)	2.10 (0.03)	1.95 (0.09)	*t* = 1.69	0.095
Neutrophil, 1,000 cells/uL, mean (SE)	4.65 (0.04)	4.59 (0.04)	4.91 (0.11)	*t* = -2.88	**0.005**
Platelet, 1,000 cells/uL, mean (SE)	233.45 (1.85)	237.36 (2.17)	217.05 (3.87)	*t* = 4.50	**<0.001**
MED score, mean (SE)	4.97 (0.06)	4.99 (0.07)	4.87 (0.09)	*t* = 1.05	0.295
Protein supply ratio, mean (SE)	15.72 (0.15)	15.74 (0.16)	15.65 (0.28)	*t* = 0.28	0.781
Carbohydrate supply ratio, mean (SE)	48.30 (0.31)	47.83 (0.35)	50.29 (0.53)	*t* = -4.15	**<0.001**
Total fat supply ratio, mean (SE)	34.42 (0.25)	34.70 (0.29)	33.23 (0.38)	*t* = 3.22	**0.002**
HB, g/dL, mean (SE)	13.78 (0.05)	13.88 (0.05)	13.33 (0.09)	*t* = 5.81	**<0.001**
Uric acid, mg/dL, mean (SE)	5.92 (0.04)	5.80 (0.04)	6.41 (0.07)	*t* = -7.29	**<0.001**
Serum vitamin D, nmol/L, mean (SE)	74.48 (1.07)	74.26 (1.20)	75.41 (1.77)	*t* = -0.57	0.569
Serum Na, *n* (%)				*χ* ^2^ = 5.343	0.069
Hypernatremia	85 (2.14)	69 (2.23)	16 (1.76)		
Hyponatremia	162 (4.37)	114 (3.92)	48 (6.26)		
Normal	3,431 (93.49)	2,676 (93.85)	755 (91.97)		
TSAT, *n* (%)				*χ* ^2^ = 61.011	**<0.001**
≤20	227 (6.48)	212 (7.55)	15 (1.98)		
>20	517 (14.77)	485 (17.52)	32 (3.20)		
Unknown	2,934 (78.76)	2,162 (74.92)	772 (94.82)		
Ferritin, *n* (%)				*χ* ^2^ = 112.280	**<0.001**
≤100	525 (15.40)	496 (18.20)	29 (3.68)		
>100	456 (12.37)	426 (14.64)	30 (2.86)		
Unknown	2,697 (72.23)	1,937 (67.17)	760 (93.46)		
Anemia, *n* (%)				*χ* ^2^ = 60.954	**<0.001**
No	2,876 (83.50)	2,324 (86.41)	552 (71.31)		
Yes	802 (16.50)	535 (13.59)	267 (28.69)		
Hyperuricemia, *n* (%)				*χ* ^2^ = 23.620	**<0.001**
No	2,460 (67.29)	1,989 (69.52)	471 (57.96)		
Yes	1,218 (32.71)	870 (30.48)	348 (42.04)		
Interaction between anemia and hyperuricemia, *n* (%)				*χ* ^2^ = 90.196	**<0.001**
No–no	2,004 (57.73)	1,662 (60.88)	342 (44.55)		
No–yes	872 (25.77)	662 (25.54)	210 (26.77)		
Yes–no	456 (9.56)	327 (8.65)	129 (13.41)		
Yes–yes	346 (6.93)	208 (4.94)	138 (15.28)		
Follow-up time, months, mean (SE)	63.82 (1.15)	67.14 (1.33)	49.91 (1.50)	*t* = 9.54	**<0.001**
Renal disease-specific mortality, *n* (%)					
Survival	2,859 (80.74)	2,859 (100.00)	0 (0.00)		
Died from renal disease	21 (0.50)	0 (0.00)	21 (2.62)		
Died from other cause	798 (18.76)	0 (0.00)	798 (97.38)		

CKD, chronic kidney disease; SE, standard error; PIR, poverty income ratio; MET, metabolic equivalent; CVD, cardiovascular disease; DM, diabetes mellitus; BMI, body mass index; eGFR, estimated glomerular filtration rate; UACR, urinary albumin-to-creatinine ratio; WBC, white blood cell; MED, Mediterranean; HB, hemoglobin; Na, sodium; TSAT, transferrin saturation; t, t-test; χ^2^, chi-square test. Bold values means statistically significant (P<0.05).

### Interaction between anemia and hyperuricemia on the risk of all-cause mortality in patients with CKD

To explore the roles of anemia and hyperuricemia in all-cause mortality in patients with CKD as well as the interaction between anemia and hyperuricemia, we first screened covariates associated with all-cause mortality (**Supplementary Table S2**). Then, variables that significantly linked to all-cause mortality in CKD patients were included in the multivariate models (all *P* < 0.05).

**Figure 2:**
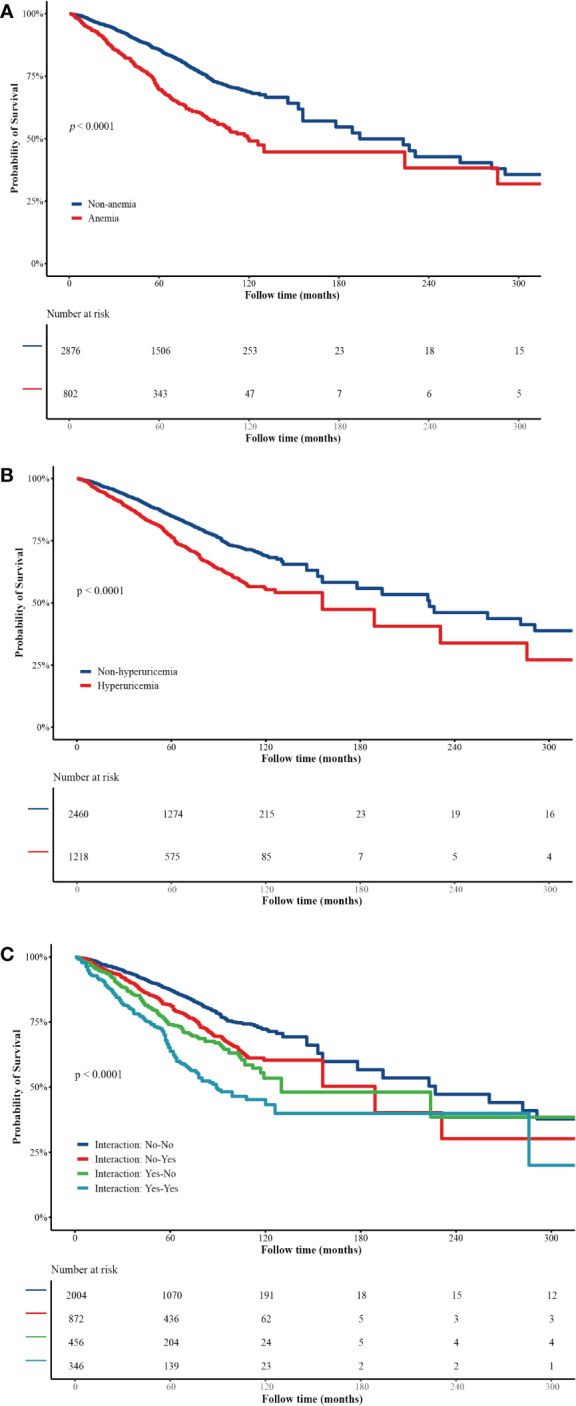
**(A)** Kaplan–Meier curves of survival probability of patients with/without anemia. **(B)** Kaplan–Meier curves of survival probability of patients with/without hyperuricemia. **(C)** Interaction effect between anemia and hyperuricemia on all-cause mortality.

**Table 2** shows the associations of anemia and hyperuricemia with all-cause mortality as well as the interaction effect between anemia and hyperuricemia on all-cause mortality. After adjusting for covariables, anemia (HR = 1.72, 95%CI: 1.42–2.09) and hyperuricemia (HR = 1.21, 95%CI: 1.01–11.45) were both linked to the risk of all-cause mortality. Compared with those who were without anemia or hyperuricemia, CKD patients with both anemia and hyperuricemia seemed to have a higher risk of all-cause mortality (HR = 2.17, 95%CI: 1.73–2.72). The results indicated that there is a potential synergetic effect between anemia and hyperuricemia on all-cause mortality, with RERI of 0.630 and AP of 0.291. In addition, the KM curves similarly showed that CKD patients with anemia or hyperuricemia had a lower survival probability than those who were without these diseases, and the potential synergetic effect between anemia and hyperuricemia was also observed (**Figure 2**).

**Table 2:** Interaction effect between anemia and hyperuricemia on the risk of all-cause mortality in patients with CKD.

Variables	Univariate model		Multivariate model^a^	
	HR (95%CI)	*P*	HR (95%CI)	*P*
Anemia				
No	Ref		Ref	
Yes	2.27 (1.88–2.74)	**<0.001**	1.72 (1.42–2.09)	**<0.001**
Hyperuricemia				
No	Ref		Ref	
Yes	1.65 (1.39–1.96)	**<0.001**	1.21 (1.01–1.45)	**0.042**
Interaction between anemia and hyperuricemia				
No–no	Ref		Ref	
No–yes	1.46 (1.22–1.75)	**<0.001**	1.07 (0.88–1.31)	0.495
Yes–no	1.97 (1.55–2.49)	**<0.001**	1.46 (1.09–1.97)	**0.012**
Yes–yes	3.53 (2.70–4.62)	**<0.001**	2.17 (1.73–2.72)	**<0.001**
RERI (95%CI)	1.100 (0.213–1.988)		0.630 (0.033–1.228)	
AP (95%CI)	0.312 (0.120–0.503)		0.291 (0.046–0.536)	

CKD, chronic kidney disease; HR, hazard ratio; CI, confidence interval; Ref, reference; RERI, relative excess risk due to interaction; AP, attributable proportion of interaction.

a Multivariate model adjusted for age, gender, race, marital status, PIR, smoking, physical activity, hypertension, CVD, DM, cancer, and carbohydrate supply ratio. Bold values means statistically significant (P<0.05).

### Interaction effect between anemia and hyperuricemia on all-cause mortality in subgroups of age, gender, CVD, hypertension, DM, and cancer

We further investigated the interaction effect between anemia and hyperuricemia on all-cause mortality in age, gender, CVD, hypertension, DM, and cancer subgroups (**Table 3**). Patients who had both anemia and hyperuricemia, compared with those who have none of these two diseases, had an increased risk of all-cause mortality no matter their age, gender, and having CVD, hypertension, DM, and cancer or not (all HR >1 and *P <*0.05). Moreover, the potential synergetic effect between anemia and hyperuricemia on all-cause mortality was only found in ≥65 years old (AP = 0.330), male (AP = 0.355), hypertension (AP = 0.736), non-hypertension (AP = 0.281), DM (AP = 0.371), and cancer (AP = 0.391) subgroups.

**Table 3:** Interaction between anemia and hyperuricemia in subgroups of age, gender, CVD, hypertension, DM, and cancer.

Subgroups	Interaction between anemia and hyperuricemia	HR (95%CI)	*P*
Age <65 years old (*n* = 1,801)	No–no	Ref	
	No–yes	1.48 (0.95 to 2.31)	0.082
	Yes–no	2.08 (1.06 to 4.08)	**0.033**
	Yes–yes	2.59 (1.30 to 5.19)	**0.008**
	RERI (95%CI)	0.027 (-1.890 to 1.944)	
	AP (95%CI)	0.010 (-0.725 to 0.746)	
Age ≥65 years old (*n* = 1,877)	No–no	Ref	
	No–yes	0.97 (0.78 to 1.21)	0.785
	Yes–no	1.40 (1.01 to 1.93)	**0.045**
	Yes–yes	2.04 (1.59 to 2.61)	**<0.001**
	RERI (95%CI)	0.672 (0.114 to 1.230)	
	AP (95%CI)	0.330 (0.083 to 0.576)	
Female (*n* = 1,855)	No–no	Ref	
	No–yes	1.09 (0.79 to 1.52)	0.595
	Yes–no	1.42 (0.91 to 2.22)	0.118
	Yes–yes	2.07 (1.47 to 2.90)	**<0.001**
	RERI (95%CI)	0.552 (-0.390 to 1.493)	
	AP (95%CI)	0.267 (-0.143 to 0.676)	
Male (*n* = 1,823)	No–no	Ref	
	No–yes	1.01 (0.73 to 1.39)	0.951
	Yes–no	1.56 (1.07 to 2.28)	**0.022**
	Yes–yes	2.44 (1.82 to 3.27)	**<0.001**
	RERI (95%CI)	0.865 (-0.126 to 1.857)	
	AP (95%CI)	0.355 (0.009 to 0.701)	
Non-hypertension (*n* = 669)	No–no	Ref	
	No–yes	0.90 (0.33–2.44)	0.837
	Yes–no	1.95 (0.64–5.96)	0.239
	Yes–yes	7.00 (1.67–29.35)	**0.008**
	RERI (95%CI)	5.155 (-3.476 to 13.785)	
	AP (95%CI)	0.736 (0.407 to 1.065)	
Hypertension (*n* = 3,009)	No–no	Ref	
	No–yes	1.06 (0.85 to 1.32)	0.584
	Yes–no	1.45 (1.07 to 1.95)	**0.016**
	Yes–yes	2.10 (1.66 to 2.67)	**<0.001**
	RERI (95%CI)	0.592 (0.004 to 1.179)	
	AP (95%CI)	0.281 (0.030 to 0.533)	
Non-CVD (*n* = 2,010)	No–no	Ref	
	No–yes	0.90 (0.63 to 1.29)	0.558
	Yes–no	1.38 (0.78 to 2.45)	0.265
	Yes–yes	2.27 (1.64 to 3.15)	**<0.001**
	RERI (95%CI)	0.989 (-0.193 to 2.170)	
	AP (95%CI)	0.435 (-0.011 to 0.882)	
CVD (*n* = 1,668)	No–no	Ref	
	No–yes	1.16 (0.87 to 1.56)	0.305
	Yes–no	1.48 (1.06 to 2.06)	**0.023**
	Yes–yes	2.09 (1.57 to 2.77)	**<0.001**
	RERI (95%CI)	0.445 (-0.243 to 1.133)	
	AP (95%CI)	0.213 (-0.097 to 0.524)	
Non-DM (*n* = 2,198)	No–no	Ref	
	No–yes	0.94 (0.72 to 1.24)	0.679
	Yes–no	1.62 (1.14 to 2.29)	**0.007**
	Yes–yes	1.74 (1.26 to 2.40)	**<0.001**
	RERI (95%CI)	0.178 (-0.540 to 0.896)	
	AP (95%CI)	0.102 (-0.295 to 0.500)	
DM (*n* = 1,480)	No–no	Ref	
	No–yes	1.28 (0.94 to 1.76)	0.116
	Yes–no	1.43 (0.89 to 2.29)	0.134
	Yes–yes	2.73 (1.88 to 3.95)	**<0.001**
	RERI (95%CI)	1.013 (-0.033 to 2.059)	
	AP (95%CI)	0.371 (0.059 to 0.683)	
Non-cancer (*n* = 2,972)	No–no	Ref	
	No–yes	1.19 (0.94 to 1.52)	0.152
	Yes–no	1.58 (1.08 to 2.30)	**0.018**
	Yes–yes	2.37 (1.81 to 3.09)	**<0.001**
	RERI (95%CI)	0.591 (-0.146 to 1.329)	
	AP (95%CI)	0.250 (-0.040 to 0.540)	
Cancer (*n* = 613)	No–no	Ref	
	No–yes	0.85 (0.58 to 1.24)	0.402
	Yes–no	1.25 (0.80 to 1.97)	0.324
	Yes–yes	1.81 (1.17 to 2.80)	**0.008**
	RERI (95%CI)	0.708 (-0.069 to 1.486)	
	AP (95%CI)	0.391 (0.039 to 0.742)	

Age subgroups adjusted for gender, race, marital status, PIR, smoking, physical activity, hypertension, CVD, DM, cancer, and carbohydrate supply ratio. Gender subgroups adjusted for age, race, marital status, PIR, smoking, physical activity, hypertension, CVD, DM, cancer, and carbohydrate supply ratio. Hypertension subgroups adjusted for age, gender, race, marital status, PIR, smoking, physical activity, CVD, DM, cancer, and carbohydrate supply ratio. CVD subgroups adjusted for age, gender, race, marital status, PIR, smoking, physical activity, hypertension, DM, cancer, and carbohydrate supply ratio. DM subgroups adjusted for age, gender, race, marital status, PIR, smoking, physical activity, hypertension, CVD, cancer, and carbohydrate supply ratio. Cancer subgroups adjusted for age, gender, race, marital status, PIR, smoking, physical activity, hypertension, CVD, DM, and carbohydrate supply ratio.

CVD, cardiovascular disease; DM, diabetes mellitus; HR, hazard ratio; CI, confidence interval; Ref, reference; RERI, relative excess risk due to interaction; AP, attributable proportion of interaction.

No–no, non-anemia and non-hyperuricemia; No–yes, non-anemia and hyperuricemia; Yes–no, anemia and non-hyperuricemia; Yes–yes, anemia and hyperuricemia. Bold values means statistically significant (P<0.05).

## Discussion

In the current study, we explored the interaction effect between anemia and hyperuricemia on the risk of all-cause mortality in patients with CKD. We found that patients with both anemia and hyperuricemia combined had a higher risk of all-cause mortality. Patients who have these two diseases seemed to have a higher risk of all-cause mortality compared with those who were without anemia or hyperuricemia, suggesting a potential synergetic effect between anemia and hyperuricemia on all-cause mortality. Besides this, this potential synergetic effect was also found in ≥65 years old, male, hypertension, non-hypertension, DM, and cancer subgroups.

As a common complication of CKD, anemia is associated with an increased risk of CKD progression as well as all-cause mortality. In a longitudinal cohort among a population with CKD, researchers reported a higher baseline prevalence of anemia in patients who died than in those who survived (25). Thorp et al. (26) evaluated patients with CKD from a large health maintenance organization administrative data set and found that anemia may be a predictor of excess mortality and excess end-stage renal disease. The relationship between anemia and all-cause mortality was also found in another prospective cohort of patients with CKD stages 3–5, which was related to the severity of anemia (27). Similarly, in the current study, having anemia was associated with a high risk of all-cause mortality in patients with CKD. The potential mechanisms involved in the association between anemia and CKD are various and complex, including the decreased production of endogenous erythropoietin (EPO), deficiency of absolute and/or functional iron, and inflammation with the hepcidin levels increased (28). Diverse medications have been used for the treatment and management of anemia in CKD. Currently, the standard of care includes supplementation of iron (oral or intravenous) erythropoiesis-stimulating agents, and RBC transfusion (29). In our study, there was a total of 802 patients who had anemia, and we also compared the serum TAST and ferritin levels of CKD patients between the survival group and the all-cause mortality group, which reflected the iron reserve status in patients (30). However, most of the participants’ information on serum TAST or ferritin were “unknown”; thus, the actual status of the iron reserve in CKD patients has not been clarified to assess their treatments, which may influence the risk of mortality.

Hyperuricemia is associated with all-cause mortality both in the general population (31, 32) and in patients with CKD (33, 34). Yang et al. (33) performed a multicenter prospective cohort study and found a U-shaped relationship between serum uric acid levels and the risk of all-cause mortality in maintenance hemodialysis patients. However, a systematic review and meta-analysis conducted by Zhang et al. (34) showed that, in patients with asymptomatic hyperuricemia, urate-lowering therapy did not decay the progression of kidney function. Basing on the results of our research, hyperuricemia was linked to a high risk of all-cause mortality in patients with CKD. Serum uric acid has been shown to predict the all-cause mortality as well as the onset and progression of CKD (35, 36). In patients with kidney disease, the decrease in acid urid urinary excretion is related to increased serum uric acid levels, which may be buffered by gastrointestinal excretory compensation (37). Hyperuricemia stimulates the renin–angiotensin system through inducing renal vasoconstriction and increasing blood pressure, which result in a vicious circle leading to the progression of renal disease (38). Besides this, eGFR is unquestionably one of the main determinants of serum uric acid levels, and the results of the interaction effect regression analysis by Russo et al. (39) indicated that serum uric acid and eGFR interplay in determining mortality. Similarly, in the present study, the eGFR levels between the survival group and the all-cause mortality group were significantly different (87.15 vs. 64.57 mL/min/1.73 m^2^). Nevertheless, Gnemmi et al. (40) implied that asymptomatic hyperuricemia acts as an anti-oxidant on macrophages and tubular epithelial cells, which endorses the recovery of kidney function and structure upon acute kidney injury. Moreover, Gonzalez-Martin et al. (41) considered that too much urate lowering can potentially increase all-cause mortality in patients with CKD. The specific mechanism that hyperuricemia was linked to all-cause mortality in patients with CKD is still unclear, which needed to be further clarified.

A potential synergetic effect between anemia and hyperuricemia on all-cause mortality in CKD patients has been observed. To the best of our knowledge, no studies have explored this interaction effect on all-cause mortality in CKD patients. The follow-up study in a Taiwan population showed that anemia and hyperuricemia were linked to new-onset CKD, respectively, and also had a synergetic effect on new-onset CKD (19). Compared with their study, our study population was from the NHANES database, which includes the representative populations in the United States. We also found a potential synergetic effect between anemia and hyperuricemia on all-cause mortality, with RERI of 0.630 and AP of 0.291 after adjusting for the covariables. The roles of anemia and hyperuricemia in CKD are similar, which can induce oxidative stress and inflammation in the extracellular matrix and endothelial cells, further leading to glomerular hypertension and sclerosis (17, 18). Then, it could impair blood flow to the kidneys, increase vascular resistance in the kidneys, and result in a decline in eGFR. In addition, a decline in renal function, in turn, may progressively impair the metabolism and clearance of uric acid and anemia. These explanations could be applied in our findings that the potential synergistic effect between anemia and hyperuricemia may further impair renal function and even mortality. Moreover, the sample size of the current study was smaller than that of Chen’s (19). A large-sample prospective cohort study on the synergistic effect between anemia and hyperuricemia on the risk of all-cause mortality in CKD patients is necessary.

The potential synergetic effect between anemia and hyperuricemia was also found in subgroups of age, gender, hypertension, DM, and cancers. Aging and hypertension are recognized risk factors for mortality, and aging is characterized by progressive structural and functional changes in the kidney as well as in the cardiovascular system, leading to a decline in renal function and hypertension (42). Clinicians are required to summarize the most appropriate therapeutic approaches in individuals over 65 years old with CKD or had both hypertension and CKD to reduce the burdens relevant to medical, social, and economic impact. Studies have reported that male patients had a higher risk of progression, a steeper eGFR decline, and a higher risk of mortality prior to kidney replacement therapy compared with female patients (43, 44). The reason for these potential sex-related differences in the progression and prognosis of CKD is unclear. However, differences in underlying risk factors and the effect of sex hormones have been proposed (45)—for example, animal studies have shown protective, anti-inflammatory responses of estrogens on podocytes and permeability-reducing effects in glomerular endothelium (46, 47). A retrospective cohort study on 4,380 patients with CKD stages 3–5 during a 7-year period found that DM and hyperuricemia are both strongly related to higher all-cause mortality and end-stage kidney disease risk, and when DM and hyperuricemia occurred together, this could further increase both the all-cause mortality and end-stage kidney disease risks (48). DM and hyperuricemia share some similar mechanisms for renal injury in patients with CKD, and high serum uric acid levels potentiate CKD progression in patients with type 2 diabetes (T2D) (49). Our results additionally showed a synergetic effect between anemia and hyperuricemia on all-cause mortality in CKD patients with DM. Patients with cancer as well as some nonmalignant hematologic disorders may receive life-saving therapy: hematopoietic stem cell transplantation. Stem cell transplantation has been reported to be associated with kidney injury directly (a wide range of structural and functional abnormalities) (50). The study results implied that attention should be paid on the synergetic effect between anemia and hyperuricemia among CKD patients combined with cancers in order to reduce the risk of mortality.

The present study was the first to discuss the synergetic effect between anemia and hyperuricemia on risk of all-cause mortality in patients with CKD, and the results may provide some reference for the management of CKD patients who had a high risk of mortality. However, there were also some limitations. Information on ferritin had too much missing data, which may block the exploration for the effects of different types of anemia on survival in CKD. Although numerous covariates were considered, data from the investigation to the end of follow-up were unavailable in the NHANES, which may result in some bias in the results. Additionally, the participants in the NHANES database were relatively representative of the population in the United States, but the applicability of these study results to populations in other countries and regions needs to be further verified.

## Conclusion

Anemia and hyperuricemia were both linked to all-cause mortality in patients with CKD, and there was a potential synergetic effect between them on the risk of all-cause mortality. Further studies are needed to clarify the causal relationships between this synergetic and mortality in CKD.

## Data availability statement

The original contributions presented in the study are included in the article/**Supplementary Material**. Further inquiries can be directed to the corresponding author.

## Ethics statement

The requirement of ethical approval was waived by Blood Purification Center, Beijing Puren Hospital for the studies involving humans because the data was accessed from NHANES (a publicly available database). The studies were conducted in accordance with the local legislation and institutional requirements. The ethics committee/institutional review board also waived the requirement of written informed consent for participation from the participants or the participants’ legal guardians/next of kin because retrospective nature of the study.

## Author contributions

ZL: Writing – review & editing, Writing – original draft, Supervision, Project administration, Conceptualization. FL: Writing – review & editing, Methodology, Investigation, Formal analysis, Data curation. RZ: Writing – review & editing, Methodology, Investigation, Formal analysis, Data curation. SG: Writing – review & editing, Methodology, Investigation, Formal analysis, Data curation.
