# Supplementary material for: Interaction between anemia and hyperuricemia in the risk of all-cause mortality in patients with chronic kidney disease
Source: Front Endocrinol (Lausanne). 2024 Mar 22;15:1286206. doi: 10.3389/fendo.2024.1286206 (PMC10998448; doi:10.3389/fendo.2024.1286206)
Supplement: Supplementary file 2 [file Table_1.docx]

Table S1. Characteristics of patients with CKD before and after interpolation

| Variables | Before interpolation  (n=3678) | After interpolation  (n=3678) | Statistics | *P* |
| --- | --- | --- | --- | --- |
| BMI, kg/m^2^, Mean (S.E) | 30.44 (0.19) | 30.44 (0.19) | t=-0.35 | 0.724 |
| PIR, Mean (S.E) | 2.64 (0.05) | 2.62 (0.05) | t=-1.63 | 0.106 |
| Lymphocyte, 1000 cells/uL, Mean (S.E) | 2.07 (0.03) | 2.07 (0.03) | t=-1.20 | 0.233 |
| Neutrophil, 1000 cells/uL, Mean (S.E) | 4.66 (0.04) | 4.65 (0.04) | t=-1.59 | 0.117 |

CKD: chronic kidney disease, SE: standard error, PIR: poverty income ratio, BMI: body mass index

t: t test

Table S2. Covariates related to all-cause mortality in patients with CKD

| Variables | Model 1 | | Model 2 | |
| --- | --- | --- | --- | --- |
|  | HR (95% CI) | *P* | HR (95% CI) | *P* |
| Age |  |  |  |  |
| < 65 | Ref |  | Ref |  |
| ≥ 65 | 4.45 (3.46-5.73) | <0.001 | 2.48 (1.85-3.32) | <0.001 |
| Gender |  |  |  |  |
| Female | Ref |  | Ref |  |
| Male | 1.24 (1.04-1.49) | 0.017 | 1.29 (1.04-1.58) | 0.019 |
| Race |  |  |  |  |
| White | Ref |  | Ref |  |
| Black | 0.58 (0.47-0.73) | <0.001 | 0.64 (0.50-0.82) | <0.001 |
| Others | 0.45 (0.36-0.56) | <0.001 | 0.54 (0.43-0.69) | <0.001 |
| Education level |  |  |  |  |
| Some college or above | Ref |  |  |  |
| High school graduate or below | 1.33 (1.15-1.53) | <0.001 |  |  |
| Unknown | 0.16 (0.06-0.42) | <0.001 |  |  |
| Marital status |  |  |  |  |
| Married | Ref |  | Ref |  |
| Not married | 1.23 (1.05-1.43) | 0.009 | 1.30 (1.07-1.58) | 0.009 |
| Unknown | 0.13 (0.05-0.38) | <0.001 | 1.58 (0.53-4.68) | 0.407 |
| PIR | 0.88 (0.83-0.93) | <0.001 | 0.87 (0.80-0.94) | <0.001 |
| Smoking |  |  |  |  |
| No | Ref |  | Ref |  |
| Yes | 1.52 (1.28-1.81) | <0.001 | 1.26 (1.07-1.48) | 0.005 |
| Unknown | 0.31 (0.11-0.84) | 0.022 | 3.81 (1.32-10.98) | 0.014 |
| Drinking |  |  |  |  |
| Light drinking | Ref |  |  |  |
| Excessive drinking | 0.92 (0.56-1.50) | 0.737 |  |  |
| Never drinking | 1.30 (0.86-1.96) | 0.216 |  |  |
| Physical activity |  |  |  |  |
| < 450 | Ref |  | Ref |  |
| ≥ 450 | 0.62 (0.45-0.84) | 0.003 | 0.72 (0.52-0.99) | 0.046 |
| Unknown | 2.13 (1.69-2.68) | <0.001 | 1.58 (1.25-2.00) | <0.001 |
| Hypertension |  |  |  |  |
| No | Ref |  | Ref |  |
| Yes | 4.55 (3.07-6.73) | <0.001 | 1.65 (1.04-2.61) | 0.034 |
| Dyslipidemia |  |  |  |  |
| No | Ref |  |  |  |
| Yes | 1.90 (1.49-2.41) | <0.001 |  |  |
| CVD |  |  |  |  |
| No | Ref |  | Ref |  |
| Yes | 2.97 (2.47-3.57) | <0.001 | 1.49 (1.24-1.79) | <0.001 |
| DM |  |  |  |  |
| No | Ref |  | Ref |  |
| Yes | 1.82 (1.53-2.16) | <0.001 | 1.28 (1.05-1.56) | 0.016 |
| Cancer |  |  |  |  |
| No | Ref |  | Ref |  |
| Yes | 2.26 (1.85-2.75) | <0.001 | 1.37 (1.14-1.66) | 0.001 |
| Unknown | 0.14 (0.05-0.39) | <0.001 | 0.15 (0.05-0.43) | <0.001 |
| BMI |  |  |  |  |
| Underweight/Normal | Ref |  |  |  |
| Overweight | 0.99 (0.78-1.27) | 0.980 |  |  |
| Obesity | 0.94 (0.75-1.18) | 0.608 |  |  |
| WBC | 1.00 (0.96-1.04) | 1.000 |  |  |
| Lymphocyte | 0.85 (0.59-1.25) | 0.410 |  |  |
| Neutrophil | 1.00 (0.94-1.06) | 1.000 |  |  |
| Platelet | 0.99 (0.99-0.99) | <0.001 |  |  |
| MED score | 0.98 (0.94-1.02) | 0.280 |  |  |
| Protein supply ratio | 0.99 (0.97-1.02) | 0.565 |  |  |
| Carbohydrate supply ratio | 1.01 (1.01-1.02) | 0.004 | 1.01 (1.01-1.02) | 0.004 |
| Total fat supply ratio | 0.99 (0.98-0.99) | 0.155 |  |  |
| Serum vitamin D | 1.01 (1.01-1.01) | 0.063 |  |  |
| Serum Na |  |  |  |  |
| Normal | Ref |  |  |  |
| Hypernatremia | 1.54 (0.87-2.73) | 0.136 |  |  |
| Hyponatremia | 1.54 (1.06-2.25) | 0.025 |  |  |
| TSAT |  |  |  |  |
| ≤ 20 | Ref |  |  |  |
| > 20 | 0.67 (0.27-1.67) | 0.380 |  |  |
| Unknown | 0.90 (0.38-2.17) | 0.819 |  |  |
| Ferritin |  |  |  |  |
| ≤ 100 | Ref |  |  |  |
| > 100 | 1.80 (0.96-3.35) | 0.065 |  |  |
| Unknown | 3.28 (2.06-5.23) | <0.001 |  |  |

CKD: chronic kidney disease, HR: hazard ratio, CI: confidence interval, Ref: reference, PIR: poverty income ratio, CVD: cardiovascular disease, DM: diabetes mellitus, BMI: body mass index, WBC: white blood cell, MED: Mediterranean, Na: sodium, TSAT: transferrin saturation

Model 1 used the univariate COX regression analysis

Model 2 used the stepwise regression analysis
